# Imergard^TM^WP: A Non-Chemical Alternative for an Indoor Residual Spray, Effective against Pyrethroid-Resistant *Anopheles gambiae* (s.l.) in Africa

**DOI:** 10.3390/insects11050322

**Published:** 2020-05-23

**Authors:** Jean M. Deguenon, Roseric Azondekon, Fiacre R. Agossa, Gil G. Padonou, Rodrigue Anagonou, Juniace Ahoga, Boris N’dombidje, Bruno Akinro, David A. Stewart, Bo Wang, David Gittins, Larissa Tihomirov, Charles S. Apperson, Marian G. McCord, Martin C. Akogbeto, R. Michael Roe

**Affiliations:** 1 Department of Entomology and Plant Pathology, Campus Box 7647, 3230 Ligon Street, North Carolina State University, Raleigh, NC 27695, USA; jdeguen@ncsu.edu (J.M.D.); apperson@ncsu.edu (C.S.A.); 2 Centre de Recherche Entomologique de Cotonou (CREC), Cotonou 06BP2604, Benin; roseric_2000@yahoo.fr (R.A.); rofargossa@yahoo.fr (F.R.A.); pagergil@yahoo.fr (G.G.P.); rodrigue.anagonou@yahoo.fr (R.A.); meakim2015@yahoo.fr (J.A.); incas05@yahoo.fr (B.N.); akinrobruno@gmail.com (B.A.); akogbetom@yahoo.fr (M.C.A.); 3 Imerys Filtration Minerals, Inc., Roswell, GA 30076, USA; David.Stewart@imerys.com (D.A.S.); B.Wang@activeminerals.com (B.W.); David.Gittins@imerys.com (D.G.); larisa.tihomirov@imerys.com (L.T.); 4 College of Natural Resources, Campus Box 8001, 2820 Faucette Drive, North Carolina State University, Raleigh, NC 27695, USA; mmccord@ncsu.edu

**Keywords:** mosquito, *Anopheles gambiae* (s.l.), malaria, Imergard^TM^WP, mechanical insecticide, pyrethroid resistance, Africa, Benin, residual wall spray

## Abstract

Malaria is the deadliest mosquito-borne disease and kills predominantly people in sub-Saharan Africa (SSA). The now widespread mosquito resistance to pyrethroids, with rapidly growing resistance to other insecticide classes recommended by the World Health Organization (WHO), may overturn the successes gained in mosquito control in recent years. It is of utmost importance to search for new, inexpensive, and safe alternatives, with new modes of action, that might improve the efficacy of current insecticides. The efficacy of a novel mechanical insecticidal mineral derived from volcanic rock, Imergard^TM^WP, was investigated to determine its efficacy as a stand-alone residual wall spray and as a mixture with deltamethrin (K-Othrine^®^ Polyzone) in experimental huts in Cove, Benin. The evaluation was conducted with susceptible (Kisumu) and wild-type *Anopheles gambiae* (s.l.). Deltamethrin applied alone demonstrated 40–45% mortality (at 72 h post-exposure) during the first four months, which declined to 25% at six months for wild *An. gambiae* from Cove. Imergard^TM^WP alone and mixed with deltamethrin, under the same assay conditions, produced 79–82% and 73–81% mortality, respectively, during the same six-month period. Imergard^TM^WP met the 80% WHO bio-efficacy threshold for residual activity for the first five months with 78% residual activity at six months. Imergard^TM^WP can be used as a mixture with chemical insecticides or as a stand-alone pesticide for mosquito control in Africa.

## 1. Introduction

Malaria continues to be a major life-threatening vector-borne disease globally but more so in developing countries. According to the World Health Organization (WHO), over 90% of the 228 million malaria cases and 405,000 deaths observed in 2018 were recorded in Africa [1]. It is the first cause of mortality and morbidity in West Africa, and the majority of malaria-endemic countries are in sub-Saharan Africa (SSA), which shares 80% of the global malaria burden [1,2]. Malaria parasites are transmitted by infected Anopheline mosquito species. Vector control is therefore essential in the fight against malaria and is commonly accomplished through the use of insecticide-treated nets (ITNs) and indoor residual spraying (IRS) [3]. The latter consists of applying insecticides to interior walls of a house.

IRS is highly effective in controlling malaria vectors and has contributed significantly to the successes obtained in the fight against the disease in recent years [4,5]. However, insecticide resistance is now widespread for pyrethroids, one the most used insecticide classes in vector control [6,7,8]. More concerning is that resistance to the other classes of insecticides approved by the WHO (carbamates, organochlorines, and organophosphates) has started to rise and has now been suspected or documented in several countries worldwide [6,9,10,11]. It is therefore of utmost importance to search for alternatives that not only exhibit new modes of action, but are also safer, cost-effective, sustainable, naturally occurring if possible, and compatible with the synanthropic nature of mosquitoes.

Mechanical insecticides (MIs) are minerals that come into contact with insects and produce a lethal effect. MIs exhibit insecticidal properties through a physical mode of action and have consistently been shown to control numerous agricultural pests, i.e., Coleoptera [12], Thysanoptera [13], Lepidoptera [14], and Hemiptera [15]. Their potential use in public heath, on the other hand, has received minimal attention and was almost exclusively limited to laboratory trials [16,17].

In this study, we demonstrated the efficacy of a new mineral-based mosquitocide derived from volcanic rock, Imergard^TM^WP (expanded perlite, 100%), in commercialization by the company, Imerys. Perlite is an aluminosilicate volcanic glass with a relatively high-water content, typically formed by the hydration of obsidian. It occurs naturally and has the unusual property of greatly expanding in volume under extreme temperature. Because of its low density, perlite is used as insulation or in plant growth media. Perlite is listed in the US Food and Drug Administration (FDA) database as Generally Recognized as Safe (GRAS), is classified as a feed additive by the European Food Safety Authority (EFSA), and is found in products like toothpaste [18]. It has not been considered before for vector control. The MI described in this paper (applied by spraying in water) provides a new mode of insecticide action for mosquito and malaria control, disrupting insect water balance. The current thinking is that mechanical insecticides disrupt the protective lipid layer of the insect’s cuticle, thus leading to death by desiccation (a non-toxic, i.e., non-systemic mode of action) [16,17,19]. Because the MI is an industrial mineral, its activity does not degrade with time as long as the mechanical insecticide is present and can transfer to the insect [19]. Since mechanical insecticides have not been used before in mosquito control, it should be effective against pyrethroid-resistant mosquitoes in Africa.

We evaluated the toxicity and residual activity of Imergard^TM^WP alone and as a mixture with deltamethrin for the control of susceptible and wild-type pyrethroid-resistant mosquitoes under field conditions in Africa. We demonstrated that the MI is a suitable alternative to a chemical insecticide currently in use for mosquito control and offers a long-lasting protection against mosquitoes such as the African malaria mosquito, *Anopheles gambiae* (s.l.).

## 2. Materials and Methods

### 2.1. Study Site and Experimental Huts

The study was conducted in the experimental hut station of Cove, southern Benin (7°14′ N, 2°18′ E) for six months (July to December 2017). The rice-growing fields surrounding the station provide year-round and prolific breeding sites for *An. gambiae* (s.l.). The local *Anopheles* populations are highly resistant to pyrethroids as shown from studies conducted in 2013–2014 [20] and confirmed in 2015 [21,22]. Eight experimental huts of WHO-approved West African design were used [23]. The huts were concrete experimental huts with corrugated iron roofs. The ceilings were polyethylene sheeting covered with palm thatch, and the interior walls were plastered with cement. Four window slits (1 cm gap, 2 on the front, 2 on the sides) allowed entrance of host-seeking mosquitoes but impeded their exit, forcing them to fly towards the veranda trap on the back of the hut. A schematic of the hut can be found in Hougard et al. [24].

### 2.2. Insecticide Treatments

Four treatments were evaluated, each randomly applied (2 huts per treatment, 8 huts in total) as follows: (i) unsprayed, control huts, (ii) deltamethrin 62.5 SC-PE (K-Othrine^®^ Polyzone; Bayer, Monheim am Rhein, Germany) at 25 mg/m^2^, (iii) Imergard^TM^WP (also referred to as ImG in this paper) (Imerys, Roswell, GA, USA) at 8 g/m^2^ as a wettable powder, and (iv) deltamethrin 62.5 SC-PE at 25 mg/m^2^ + Imergard^TM^WP at 4 g/m^2^. The MI was applied as a wettable powder per manufacturer’s recommendations. Maximum safety instructions and protective measures were observed as a standard practice for all treatments [22]. The huts (walls and ceiling) were sprayed using a 10L Hudson Xpert compression sprayer (Hudson Xpert, Chicago, IL, USA). A mixture was conducted to test potential potentiation between Imergard^TM^WP and a chemical insecticide.

### 2.3. Sleepers and Mosquito Collection

Eight consenting human (adult) volunteers were locally recruited and served as sleepers (mosquito collectors). They were rotated between huts on successive nights to simulate a Latin square design. They slept in the huts from 21:00 to 6:00 each night. Each morning (from 6:00), mosquitoes were collected from the huts using a mouth aspirator (main room and veranda) and transferred to the laboratory where they were identified using appropriate identification keys [25]. They were also scored as dead or alive and as fed or unfed. Mosquitoes were scored as dead if they did not move after being touched with a blunt probe. Mosquitoes were considered fed if the abdomen was visually engorged with blood. Live mosquitoes were kept in small cups, and delayed mortality was recorded for up to 72 h for all four treatments. Because the putative mode of action of the MI is desiccation (as mentioned earlier) and preliminary studies suggested that providing water sources in large cage tests had no impact on mortality, mosquitoes were not provided with a sugar solution during the holding period.

The data from the two huts for each treatment were pooled together, and treatment efficacy was expressed relative to the untreated control in terms of deterrence (reduction in hut entry relative to the untreated control huts), the exophily rate (proportion of mosquitoes, relative to that of the control, that were found in the exit trap (= veranda)), blood-feeding inhibition (reduction in blood feeding compared with that of the control), and delayed mortality (percentage mortality at 24, 48, and 72 h post-collection).

### 2.4. Residual Activity of Insecticide Treatments

The residual activity of the different insecticide treatments was determined one week after treatment application and for each month of the trial using WHO cone bioassays [23]. Laboratory maintained susceptible female *An. gambiae* “Kisumu” and wild *An. gambiae* (s.l.) from Cove were tested. Ten mosquitoes (non-blood fed, 2–5 days old with *ad libitum* access to a 10% honey-in-water solution before the transfer to cones) were introduced per cone and exposed to the walls for 30 min at different heights from the hut floor (0.5, 1, 1.5, and 2 m). Mortality was then recorded at 24 and 48 h.

### 2.5. Statistical Analyses

The raw data were managed using Microsoft Excel (Version 2016, Redmond, WA, USA). The WHO bio-efficacy threshold was used for the analyses of the residual effect [23]. Statistical analyses on deterrence, exophily, and blood feeding between the control and treatment were conducted using a two-proportion *Z*-test. The 95% confidence intervals for the proportions were computed using the binomial test. Control mortality for mosquitoes entering the huts ranged from 2.4% to 4.5% at 72 h at each time point over six months. The control residual activity for the Kisumu strain in cone tests was 4.2–16% at 48 h over six months with the majority of observations <5%. The control residual activity of the wild population was 1.6–15% at 48 h with the majority <5%. Mortality rates were Abbott corrected [26]. All analyses were completed using the R statistical software (Version 3.6.1, R Foundation for Statistical Computing, Vienna, Austria) (R Development Core Team 2016) [27].

### 2.6. Ethical Clearance

The study was approved by the Ministry of Health in Benin (Comité d’Ethique Institutionnelle du CREC, Avis éthique favorable No. 03 du 07 Juillet 2017, Lettre No 231/MS/DC/SGM/DRFMT/CREC/CEI-CREC/SA du 03 Août 2017). The human volunteer sleepers gave informed consent prior to their participation in the study.

### 2.7. Abbreviations

CREC: Centre de Recherche Entomologique de Cotonou; Delta: deltamethrin; DM, deltamethrin; EFSA: European Food Safety Authority; FDA: Food and Drug Administration; GRAS: generally recognized as safe; ImG: Imergard^TM^WP; IRS: indoor residual spraying; ITNs: insecticide-treated nets; MI: mechanical insecticide; NCSU: North Carolina State University; PE: polymer enhanced; SEM: scanning electron microscopy; SC: suspension concentrate; SE: standard error; WG: water dispersible granules; WHO: World Health Organization; WP: wettable powder.

### 2.8. Data Availabity

All of the data supporting the findings are presented in the paper.

## 3. Results

**Figure 1:**
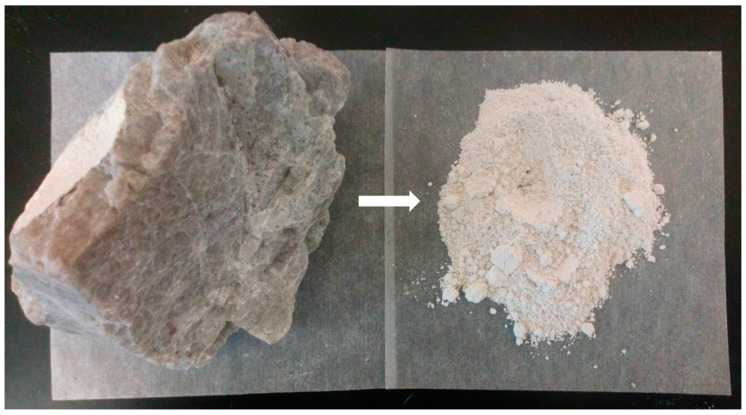
Imergard^TM^WP before and after processing. Perlite, the active ingredient of Imergard^TM^WP, is a volcanic rock (**left**) that can be expanded (when heated to 760–980 °C) and then processed (drying and grinding) to obtain the fine white powder used in this study (**right**).

**Figure 2:**
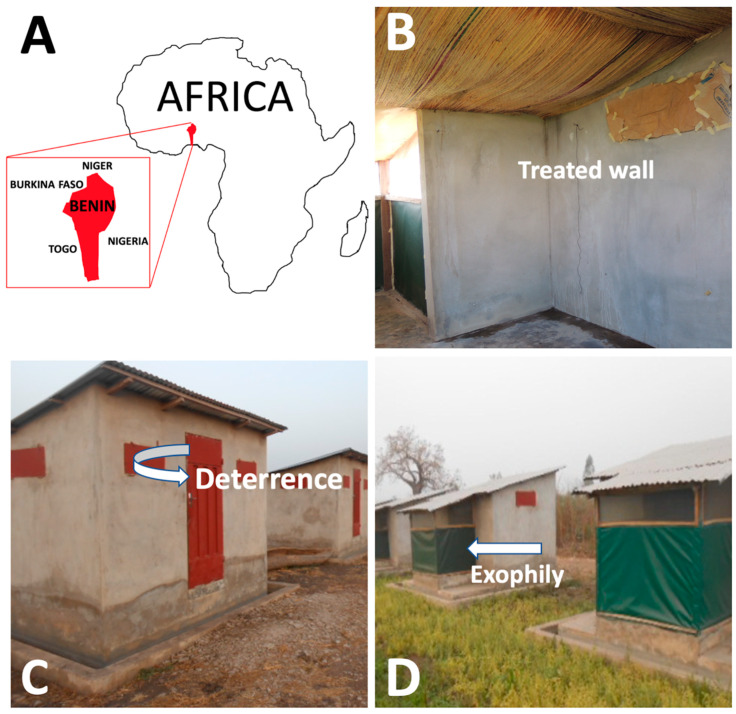
Study site and experimental huts: (**A**) location of field trial in Africa; (**B**) appearance of a treated wall; (**C**) front and side views of an experimental hut displaying the window slits for mosquito entrance as well as the movement of deterred mosquitoes; and (**D**) back view of a hut displaying the veranda trap as well as the exophily movement of mosquitoes that had entered the hut.

The efficacy of ImG (Figure 1) was evaluated in small-scale field trials in southern Benin, West Africa (Figure 2A). The MI readily mixed with water with minimal agitation and remained in suspension long enough for conducting the applications without additional agitation. Standard vector control sprayers (as described in the Materials and Methods section) without modification were adequate for the application, and Figure 2B shows the MI applied at the rate of 8 g per square meter to interior hut walls and the ceiling. The product was odorless and, once applied to the wall, did not produce any obvious airborne dust. The studies were conducted using WHO-approved standard research huts (Figure 2C,D) following WHO protocols [23]. The parameters tested are presented below.

### 3.1. Deterrence

In total, 26,551 host-seeking *An. gambiae* (s.l.) were collected during the six-month trial. In general, and compared to the untreated control, all the treatments reduced mosquito entry at rates ranging between 8% and 39% (Table 1). However, this effect (Figure 2C) was highly significant (*p* < 0.0001) and more noticeable in the huts sprayed with a mixture of ImG and deltamethrin 62.5 SC-PE where it lasted for six months (Table 1).

### 3.2. Exophily

All treatments elicited an increased exit behavior from the mosquitoes (Figure 2D, Table 2). On average, the proportion exiting the control huts was 31%. Exophily rates with deltamethrin 62.5 SC-PE (56% on average) and the mixture of ImG with deltamethrin 62.5 SC-PE (50%) were significantly (*p* < 0.0001) higher than the control huts. Exophily rates did not differ significantly between ImG (33%) and the control (31%) (*p* > 0.05).

### 3.3. Blood Feeding

A slight reduction in blood feeding was noticed during the 1st, 5th, and 6th months with ImG and during the 3rd, 5th, and 6th months with deltamethrin 62.5 SC-PE compared to the control (Table 3). No reduction in blood feeding was displayed by the mixture during the six-month trial (Table 3). However, it is important to point out that blood-feeding rates were in general very high across all treatments (>90%).

### 3.4. Toxicity

**Figure 3:**
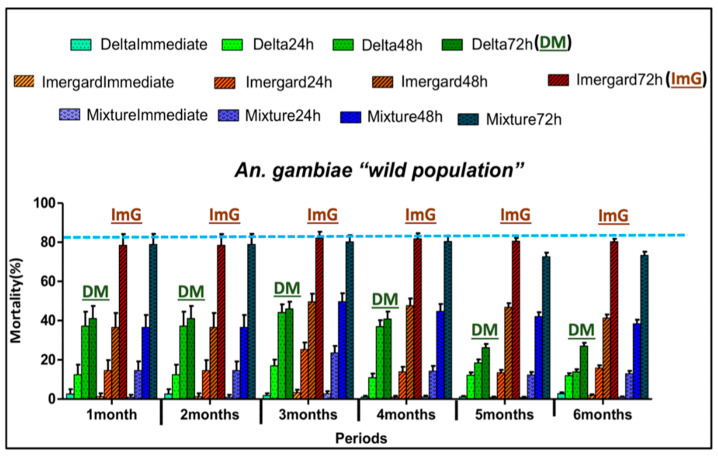
Monthly mortality rates of free-flying resistant *Anopheles gambiae* (s.l.) entering experimental huts in Cove, Benin. For each treatment, mortality was recorded at collection time (immediate) and then at 24, 48, and 72 h after collection. Error bars represent 95% confidence intervals. Delta and DM = Deltamethrin; ImG = Imergard^TM^WP; Mixture = Deltamethrin + Imergard^TM^WP. The blue dotted line indicates highest mortality rates obtained in mosquitoes collected in the huts.

Mortality rates after 72 h were low with deltamethrin 62.5 SC-PE ranging from 40% to 45% during the first four months and decreasing to about 25% for months five and six (Figure 3). On the other hand, high mortality rates were recorded over a period of six months for ImG ranging from 79% to 82%. The mixture of ImG and deltamethrin 62.5 SC-PE average mortality rate was 78%. The mortality rate for the treatments containing ImG alone, the mixture, and deltamethrin 62.5 SC-PE alone increased during the bioassay from 24 to 48 h and then from 48 to 72 h (Figure 3).

### 3.5. Insecticide Residual Activity

**Figure 4:**
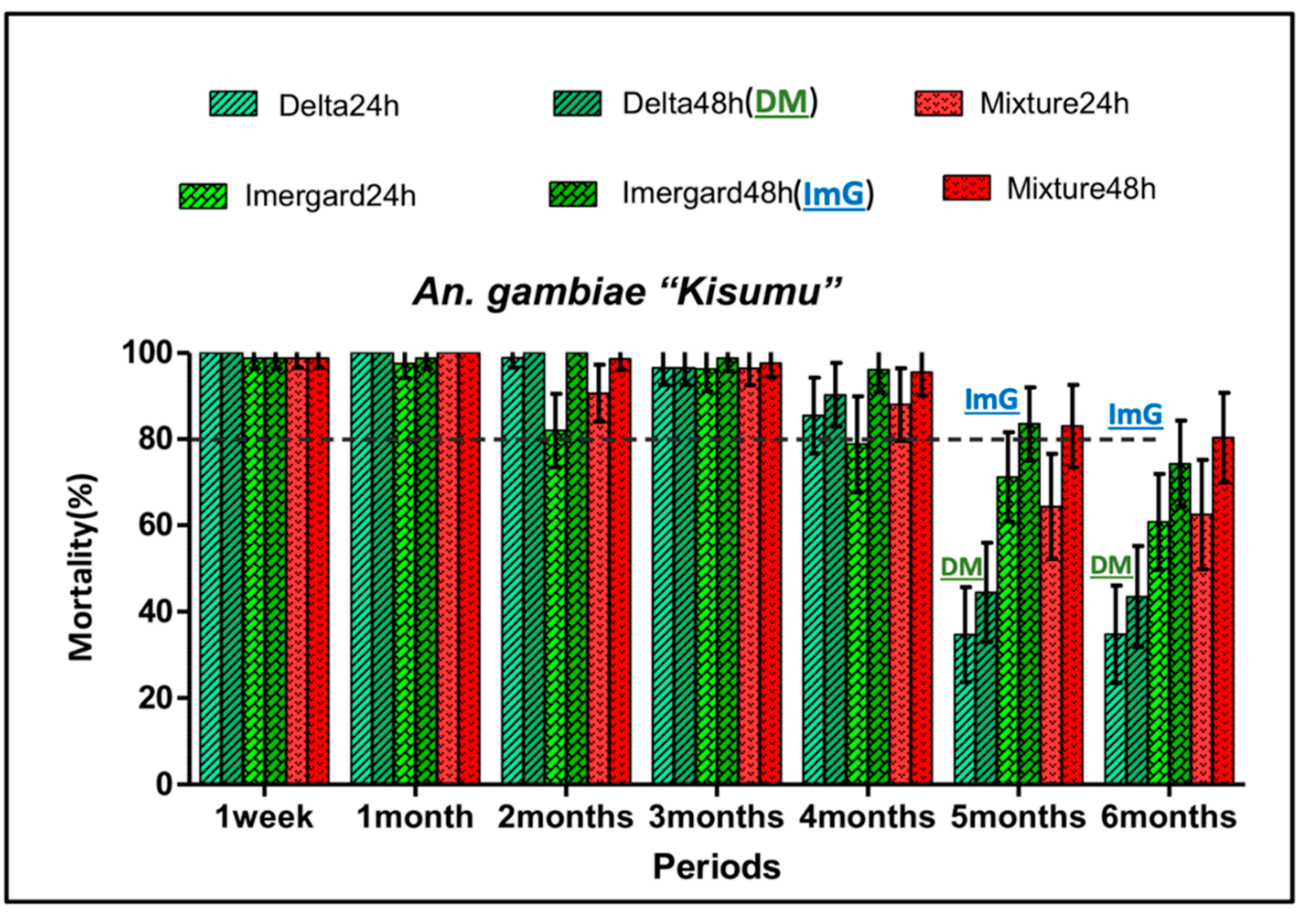
Residual mortality of Imergard^TM^WP, deltamethrin, and their mixture following cone bioassays with laboratory susceptible *Anopheles gambiae* “Kisumu” strain in experimental huts in Cove, Benin. For each treatment, mortality was recorded at 24 and 48 h after a 30 min exposure to the walls. Error bars represent 95% confidence intervals. Delta and DM = Deltamethrin; ImG = Imergard^TM^WP; Mixture = Deltamethrin + Imergard^TM^WP. The gray dotted line indicates the 80% threshold requirement of the WHO for residual activity.

**Figure 5:**
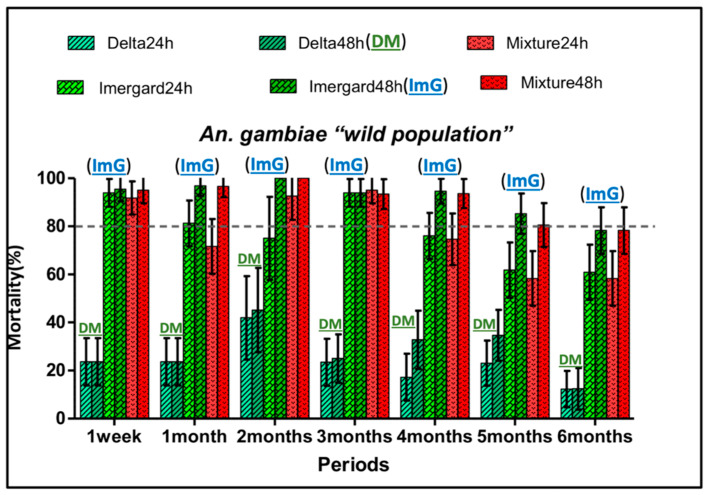
Residual mortality of Imergard^TM^WP, deltamethrin, and their mixture following cone bioassays with wild resistant *Anopheles gambiae* (s.l.) in experimental huts in Cove, Benin. For each treatment, mortality was recorded at 24 and 48 h after a 30 min exposure to the walls. Error bars represent 95% confidence intervals. Delta and DM = Deltamethrin; ImG = Imergard^TM^WP; Mixture = Deltamethrin + Imergard^TM^WP. The gray dotted line indicates the 80% threshold requirement of the WHO for residual activity.

The residual efficacy of each treatment was determined monthly by exposing pyrethroid susceptible and wild-type (pyrethroid-resistant) mosquitoes to treated walls. The WHO benchmark for bio-efficacy for a residual wall spray is 80% mortality [23]. For the susceptible “Kisumu” strain, at 48 h, the deltamethrin 62.5 SC-PE mortality was greater than 80% for the first four months and then decreased to about 45% in the last two months of the study (Figure 4). The huts treated with Imergard^TM^WP showed mortality greater than 80% for five months with a slight decrease in the last month. Mortality remained above 80% for the mixture of ImG + deltamethrin 62.5 SC-PE for the entire study (Figure 4). With the wild *An. gambiae* (s.l.) population and for the same time point (48 h), the mortality was low and never exceeded 45% with deltamethrin 62.5 SC-PE (Figure 5). With ImG and the mixture, the mortality rates remained above 80% for five months and decreased to about 78% in the sixth month (Figure 5). Imergard^TM^WP had 3.1 times greater residual activity than the deltamethrin 62.5 SC-PE alone for the first four months and had 5.7-fold greater efficacy at six months against wild-type mosquitoes. In these studies, mortality due to Imergard^TM^WP alone or as a mixture with deltamethrin 62.5 SC-PE increased during the bioassay from 24 to 48 h. These results demonstrated that an MI made from volcanic rock is a suitable alternative to deltamethrin as a residual wall spray.

## 4. Discussion

Mosquito transmitted malaria parasites kill more people each year worldwide than any other single mortality factor, especially affecting children under the age of five and expecting mothers. The WHO African region accounted for 94% of all malaria deaths in 2018, while 67% (272,000) of all malaria deaths worldwide were children under five years [1]. Vector control with chemical-based, insecticide-treated bed nets and chemical-based insecticide (residual) wall sprays is critical to mosquito management and the reduction of malaria, the former including insecticide synergists. It is estimated that the use of insecticide-treated nets and the implementation of indoor residual spraying, taken together, averted over 517 million clinical cases of malaria from 2000 to 2015 [28]. Using a mathematical model to predict the health impact of various IRS products, Sherrard-Smith et al. [29] reported that Actellic^®^ 300CS (an organophosphate) and SumiShield^®^ 50WG (a neonicotinoid) grouped together averted up to 500 clinical cases per 1000 people per year. These predictions were determined for areas with moderate endemicity, high levels of pyrethroid resistance, low bed net use and 80% IRS coverage. In Benin, two rounds of bendiocarb (a carbamate) spraying in a community trial in 2008–2009 reduced biting rates of pyrethroid-resistant *An. gambiae* by over 80% and the parous rate by 70%, while none of the mosquitoes analyzed were infected (entomological inoculation rate = 0) [30].

Since the main pesticides currently used for bed nets are pyrethroid insecticides, the use of residual wall sprays with insecticides with a different mode of action like carbamates, organophosphates, and organochlorines are essential to managing mosquito insecticide resistance to bed nets. Despite this effort, insects are increasingly becoming more resistant to pyrethroids and demonstrating cross-resistance to other insecticides. In fact, insecticide resistance in malaria vectors has been documented for pyrethroids [6,8], organophosphates and carbamates [9,31], and organochlorines [32,33]. Currently, there is a significant effort to develop new chemical insecticides with new modes of action for bed nets and residual wall sprays. As of January 2020, 30 IRS products (24 pyrethroids, 1 neonicotinoid, 2 carbamates, 2 organophosphates, and 1 dual-active ingredient neonicotinoid-pyrethroid) as well as 19 ITN kits (18 pyrethroid-based and 1 dual-active ingredient alpha-cypermethrin-chlorfenapyr) have WHO prequalification listings (https://www.who.int/pq-vector-control/prequalified-lists/en, accessed on 24 April 2020). SumiShield^®^ 50WG, for example, is a new IRS product (WHO prequalification achieved in October 2017) containing clothianidin, a neonicotinoid [22]. If alternatives are not found, we will lose mosquito control not only from wall sprays but also for bed nets, and the gains achieved in the last ten years in the reduction of malaria cases will be reversed.

The other consideration with insecticide-treated bed nets and residual wall sprays is the potential long-term use and exposure of people to chemical insecticides in the home. Contradictory results have been reported in the literature regarding the health effects of IRS insecticides, where some studies found adverse associations between exposure to insecticides such as DDT (dichlorodiphenyltrichloroethane, an organochlorine) and some pyrethroids and human health [34,35], whereas others found none [36,37]. However, the use of chemical insecticides is currently necessary because of the high risk of mortality from contracting malaria, and the insecticides being used have been proven safe. Not only are different modes of action needed for control but effective insecticides from natural sources with more specific action against the mosquito vector would be desirable.

In field trials in Benin, Africa, using WHO-approved huts (Figure 2) and standard WHO methods to evaluate residual wall sprays, ImG alone and as a mixture with deltamethrin (deltamethrin 62.5 SC-PE) was found to be significantly more effective than the pyrethroid alone and the untreated control. A deterrent effect (reduction in mosquito entry rates compared to the control) was found in the first four months (Table 1). The deterrent effect with ImG was not expected as the MI is odorless, and there was no obvious airborne MI in or exiting the experimental huts. One possible explanation is that some of the mosquitoes entering the hut made contact with a treated surface close to the hut entrance and became quiescent. When a mosquito moves, because of their proximity to the opening they have a better chance of exiting compared to the control huts. Because the entrance windows were engineered as one-way openings and deterrence levels were highly variable in different months, this explanation is most likely not the mechanism for the calculated ImG deterrence found in some months. Entry reduction rates month to month were highly variable in our study, especially for ImG, and in most cases was less than 10% when compared to the control. It is most likely that entry reductions could simply be an artifact of the random geographical positioning of the control huts used to calculate deterrence compared to the ImG-treated huts and/or could be the result of environmental differences.

In two-choice repellency studies conducted in laboratory settings at North Carolina State University (NCSU), the malaria mosquito was not repelled by an ImG-treated surface [38]. Results here showed that once mosquitoes were in the hut, Imergard^TM^WP did not repel the mosquitoes into the veranda (Table 2); there was no difference in exophily rates (the proportion of mosquitoes found in the hut veranda) between the control and the MI. This is consistent with the idea that an ImG surface is not repellent. The higher exophily rates observed with deltamethrin alone and the ImG–deltamethrin mixture is expected since pyrethroids are known to demonstrate mosquito spatial repellency [21,39].

No significant blood-feeding inhibition was observed for ImG, the pyrethroid, or the mixture of ImG with the pyrethroid (Table 3). In fact, blood-feeding rates were high in all treatments with >90% of the mosquitoes entering the huts acquiring a blood meal in both the treated and untreated huts. This is not uncommon for IRS treatments. No significant differences in blood-feeding rates were found between the treated and untreated huts during the evaluation of chlorfenapyr [21], clothianidin [20,22], and bendiocarb [40] in Benin.

Since there is no physical barrier, host-seeking mosquitoes often feed on the sleeper before landing on the walls to rest and digest their meal [20,22]. High percentages of blood-fed *An. gambiae* (s.l.) died after resting on the walls treated with Imergard^TM^WP (79–82% average mortality) and the mixture (73–81%) during the 1–6-month study (Figure 3), thus confirming this technology has a significant potential as a new active ingredient for IRS. Furthermore, its residual mean activity as a stand-alone treatment was ≥80% mortality for at least five months for both susceptible (Figure 4) and resistant (Figure 5) mosquitoes; activity at six months was 76% and 78%, respectively (although the 95% upper confidence interval exceeds 80%). Although further studies are needed, it is possible that 80% mortality could be achieved past six months using the application rate in this study and/or increased even further by increasing the application rate. Examining application rates versus control duration for ≥80% mortality is needed in the future. The use of ImG at an optimum application rate for maximum control over time could lead to a reduction in the number of applications needed each year, a reduction in IRS application costs per home, and an increase in the number of homes that could be treated with the resources currently available. Furthermore, since ImG is not subject to thermal degradation, metal roofs can be treated, further improving efficacy. Based on the differences in mortality in the huts over six months, the MI killed 2.5-fold more mosquitoes (5517) than the pyrethroid (2180).

Imergard^TM^WP showed a time-dependent increase in insecticidal activity against mosquitoes from 24 to 72 h. When a mosquito lands on a treated surface, a few particles of the product are statically transferred onto the insect’s body. The insect’s epicuticle contains lipids to prevent water loss [41,42]. Current understanding is that the lipid layer is disrupted by the mechanical insecticide. This increases water loss, disrupts the osmolality of the hemolymph, and results in death. The disruption of the lipid layer of the insect’s cuticle is the presumed mechanism of action for MIs [16,17,41], and this process can take a few hours to a couple of days depending on the MI, the insect species, dose, environmental conditions (temperature and humidity), and the time since the last consumption of water or a blood meal. This delayed effect was also noticed with non-pyrethroid insecticides in IRS trials, e.g., with clothianidin (a neonicotinoid) [20] and chlorfenapyr (a pyrrole) [21]. It is therefore important that the WHO IRS insecticide evaluation guidelines, originally conceived for fast-acting insecticides, be reviewed or adjusted to account for the varying modes of action of new IRS alternatives currently being investigated.

Mosquitoes exposed to Imergard in the residual activity tests died at a faster and higher rate than the mosquitoes collected inside the huts. In the latter, mosquitoes entered freely and, once inside the huts, had the possibility of moving to different surfaces of the huts and into the exit traps. Some of these surfaces were untreated. In the residual efficacy tests, mosquitoes were confined to a small space (WHO cones) presumably increasing their exposure to the treated surface for 30 min, and the mosquitoes may have acquired a higher amount of the insecticide in a shorter period. In laboratory studies, when *An. gambiae* were continuously exposed to Imergard in cones, 50% of the mosquitoes were dead in 5 h [38].

During the study, mosquitoes were not provided access to a 10% honey-in-water solution during the holding period. The sugar solution fed to mosquitoes used in the residual activity tests prior to their use in this study and the high blood-feeding rates (>90%) in mosquitoes collected inside the huts allowed them to survive without dying from starvation for the time course of the bioassays [43,44,45]. Blood feeding may have also decreased the rate of death during holding for the hut-collected mosquitoes. The MI could easily be washed away or become wet during prolonged interactions of the mosquito with a water source in their holding container. The close housing of mosquitoes within a few cm of a water source, comprised of a large wet surface area optimized for mosquito availability and feeding, creates artificial conditions not found under field conditions. For mechanical insecticides, water provisioning in confinement artificially blocks their mode of action. This is an important example where having rigid testing guidelines for evaluating new IRS technology requiring water for the mosquito might prevent the discovery and implementation of a new mode of action.

A legitimate concern could be that, in houses, access to wet environments might be possible and could reduce or prevent Imergard^TM^WP action. However, this is not de facto true. After blood feeding, endophilic/anthropophilic mosquitoes seek a protected resting site inside the house (or sometimes outside) where they can digest the blood meal and develop eggs. At least 50% of *An. gambiae* females stay inside the home after a blood meal and invade interior walls, crevices, furniture, etc., for that purpose [46]. This stage of the gonotrophic cycle takes 1.8 days up to several days, where the duration varies depending on the mosquito species, the amount of blood consumed, and the environmental conditions [46,47,48,49]. Because blood digestion and egg maturation occur at rest with little to no interaction with a water source, it is likely that under natural conditions in a home the MI would have produced its lethal effect before the mosquito finished blood meal digestion and began to seek an oviposition site.

One concern about the use of a mechanical insecticide on the walls of a home is that human contact with treated surfaces might prematurely reduce the duration of control. A possible solution could be to treat only the upper part of the walls or target places where mosquitoes rest, such as crevices. Djenontin et al. [50] showed that covering only the upper one-third of the wall of huts with a carbamate-treated plastic sheeting (CTPS) provided equal or better efficacy compared to a traditional IRS application method using bendiocarb.

Improved toxicity and residual activity were obtained by mixing deltamethrin with ImG compared to deltamethrin alone especially for wild-type insecticide-resistant *An. gambiae* (Figure 3, Figure 4 and Figure 5). Mixing two insecticides with different modes of actions to improve vector control even in areas of resistance was suggested before and recommended [6]; using two different modes of action could delay the evolution of resistance or reverse current levels of resistance. The WHO recommends that the two insecticides be co-formulated into a single product [6].

The poor performance observed with deltamethrin in our study (even though not surprising) is worrisome. Deltamethrin 62.5 SC-PE (a.i. 62.5 g/L deltamethrin; Bayer, WHOPES recommendations achieved in September 2013) is a polymer-enhanced suspension concentrate that came on the market only a few years ago and has been shown in several studies to be more effective than the standard deltamethrin WG25 against both susceptible and wild resistant mosquitoes [51,52]. The work here is suggesting that improving current active ingredients by formulation alone will not be sufficient in resolving resistance issues for the long term and that new active ingredients with a different mode of action are needed.

## 5. Conclusions

In summary, the use of a mechanical insecticide derived from volcanic glass represents a new paradigm for mosquito and malaria control. Applied as a stand-alone treatment or as a mixture with deltamethrin, Imergard^TM^WP provided ≥80% control for five months for both susceptible and pyrethroid-resistant *Anopheles* mosquitoes in Africa with the likelihood of even longer-term activity. Activity at six months was 76% and 78%, respectively. Due to its new mode of action (in vector control), natural origin, safety (mammalian toxicity >10 g/kg) [18], abundance in nature, and low estimated cost, ImG should be an effective non-chemical alternative to current insecticides used in IRS. Imergard^TM^WP, because of its mechanical mechanism of action, has a low probability of increasing insecticide resistance to chemical insecticides and therefore can be a powerful tool for integrated resistance management in Africa. The MI easily mixes with water and is applied using standard vector control spray equipment. This represents the first report on the practical use of a mineral in the field for mosquito control in Africa. More studies are needed to fully understand the potential of this new technology.

## Figures and Tables

**Table 1:** Hut entry reduction in wild-type *Anopheles gambiae* (s.l.).

Treatment	Months	Total ^a^	Proportion (%) ^b^	95% CI ^c^	*p*-Value ^d^
Control	1	785			
	2	331			
	3	592			
	4	796			
	5	2084			
	6	2355			
Deltamethrin	1	716	8.8	6.7–10.9	<0.0001
	2	203	38.7	33.1–44.2	<0.0001
	3	542	8.4	6.0–10.8	<0.0001
	4	838	-	-	>0.05
	5	1918	8.0	6.8–9.2	<0.0001
	6	2539	-	-	>0.05
Imergard^TM^WP	1	645	17.8	15.0–20.6	<0.0001
	2	204	38.4	32.8–43.9	<0.0001
	3	556	6.1	4.0–8.2	<0.0001
	4	719	9.7	7.5–11.8	<0.0001
	5	2098	-	-	>0.05
	6	2658	-	-	>0.05
DeltaM + ImG^e^	1	587	25.2	22.1–28.4	<0.0001
	2	257	22.4	17.6–27.2	<0.0001
	3	527	11.0	8.3–13.7	<0.0001
	4	678	14.8	12.2–17.4	<0.0001
	5	1760	15.6	13.9–17.2	<0.0001
	6	2163	8.2	7.0–9.3	<0.0001

^a^ Total number of mosquitoes collected in huts for each month indicated. ^b^ Percentage reduction of mosquitoes found in treated huts compared to the number of mosquitoes found in the control; dashes represent values less than zero. ^c^ CI, confidence interval; dashes indicate no CI was calculated. ^d^ Two-proportion *Z*-test, α = 0.05. ^e^ Deltamethrin and Imergard^TM^WP mixed together and then sprayed.

**Table 2:** Exophily rates ^a^ at different times after spraying for wild-type *An. gambiae* (s.l.).

Treatment	Months	Total ^b^	Proportion (%) ^a^	95% CI ^c^	*p*-Value ^d^
Control	1	785	26.9	23.8–30.1	-
	2	331	26.3	21.6–31.4	-
	3	592	31.6	27.9–35.5	-
	4	796	30.2	33.5–37.0	-
	5	2084	34.7	32.6–36.8	-
	6	2355	33.8	31.9–35.8	-
Deltamethrin	1	716	53.2	49.5–56.9	<0.0001
	2	203	56.2	49.0–63.1	<0.0001
	3	542	53.7	49.4–58.0	<0.0001
	4	838	56.4	53.0–59.8	<0.0001
	5	1918	59.8	57.6–62.0	<0.0001
	6	2539	57.1	55.1–59.0	<0.0001
Imergard	1	645	31.0	27.4–34.7	0.097
	2	204	34.8	28.1–41.6	0.045
	3	556	31.3	27.5–35.3	0.966
	4	719	33.2	29.8–36.8	0.216
	5	2098	33.6	31.5–35.6	0.458
	6	2658	33.5	31.7–35.3	0.858
DeltaM+ ImG ^e^	1	587	48.2	44.1–52.3	<0.0001
	2	257	48.6	42.4–54.9	<0.0001
	3	527	48.4	44.0–52.8	<0.0001
	4	678	48.7	44.8–52.5	<0.0001
	5	1760	49.9	47.2–52.0	<0.0001
	6	2163	50.2	48.0–52.3	<0.0001

^a^ Percentage of mosquitoes entering the hut that were found in the veranda. ^b^ Total number of mosquitoes collected in huts for each month indicated. ^c^ CI, confidence interval. ^d^ Two-proportion *Z*-test, α = 0.05. ^e^ Deltamethrin and Imergard^TM^WP mixed together and then sprayed.

**Table 3:** Blood-feeding rates ^a^ of wild-type *An. gambiae* (s.l.) collected in the huts.

Treatment	Months	Total ^b^	Proportion (%) ^a^	95% CI ^c^	*p*-Value ^d^
Control	1	785	94.3	92.4–95.8	-
	2	331	95.5	92.6–97.6	-
	3	592	92.4	90.0–94.4	-
	4	796	95.5	93.8–96.8	-
	5	2084	97.4	96.6–98.0	-
	6	2355	96.1	95.3–96.9	-
Deltamethrin	1	716	94.8	92.9–96.3	0.713
	2	203	97.0	93.0–99.0	0.496
	3	542	95.9	93.9–97.4	0.016
	4	838	96.7	95.2–97.8	0.270
	5	1918	94.2	93.0–95.2	<0.0001
	6	2539	92.2	91.1–93.2	<0.0001
Imergard	1	645	89.3	86.6–91.6	0.0008
	2	204	98.0	94.9–99.6	0.186
	3	556	93.4	90.9–95.3	0.612
	4	719	95.3	93.4–96.7	0.956
	5	2098	95.5	94.5–96.3	0.0013
	6	2658	91.0	90.4–92.5	<0.0001
DeltaM+ ImG ^e^	1	587	94.2	92.0–96.0	>0.05
	2	257	98.1	95.4–99. 5	0.137
	3	527	92.2	89.6–94.4	>0.05
	4	678	94.2	92.2–95.9	0.341
	5	1760	96.8	95.8–97.5	0.315
	6	2163	95.9	95.0–96.7	0.723

^a^ Percentage of mosquitoes in the hut that were found in the blood-fed state. ^b^ Total number of mosquitoes collected in huts for each month indicated. ^c^ CI, confidence interval. ^d^ Two-proportion *Z*-test, α = 0.05. ^e^Deltamethrin and Imergard^TM^WP mixed together and then sprayed.
